# Using laboratory-based surveillance data for prevention: an algorithm for detecting Salmonella outbreaks.

**DOI:** 10.3201/eid0303.970322

**Published:** 1997

**Authors:** L C Hutwagner, E K Maloney, N H Bean, L Slutsker, S M Martin

**Affiliations:** Centers for Disease Control and Prevention, Atlanta, Georgia, USA.

## Abstract

By applying cumulative sums (CUSUM), a quality control method commonly used in manufacturing, we constructed a process for detecting unusual clusters among reported laboratory isolates of disease-causing organisms. We developed a computer algorithm based on minimal adjustments to the CUSUM method, which cumulates sums of the differences between frequencies of isolates and their expected means; we used the algorithm to identify outbreaks of Salmonella Enteritidis isolates reported in 1993. By comparing these detected outbreaks with known reported outbreaks, we estimated the sensitivity, specificity, and false-positive rate of the method. Sensitivity by state in which the outbreak was reported was 0%(0/1) to 100%. Specificity was 64% to 100%, and the false-positive rate was 0 to 1.


### Using Laboratory-Based Surveillance Data for Prevention: An Algorithm for Detecting *Salmonella* Outbreaks

By applying cumulative sums (CUSUM), a quality control method commonly used in manufacturing, we constructed a process for detecting unusual clusters among reported laboratory isolates of disease-causing organisms. We developed a computer algorithm based on minimal adjustments to the CUSUM method, which cumulates sums of the differences between frequencies of isolates and their expected means; we used the algorithm to identify outbreaks of *Salmonella* Enteritidis isolates reported in 1993. By comparing these detected outbreaks with known reported outbreaks, we estimated the sensitivity, specificity, and false-positive rate of the method. Sensitivity by state in which the outbreak was reported was 0%(0/1) to 100%. Specificity was 64% to 100%, and the false-positive rate was 0 to 1.

Effective surveillance systems provide baseline information on incidence trends and geographic distribution of known infectious agents. The ability to provide such information is a prerequisite to detecting new or reemerging threats (1). Laboratory-based surveillance can provide data on the location and frequency of isolation of specific pathogens, which can be used to rapidly detect unusual increases or clusters. These data can be transmitted electronically from multiple public health sites to a central location for analysis.

Many acute outbreaks of infectious diseases are detected by astute clinical observers, local public health authorities, or the affected persons themselves. However, outbreaks dispersed over a broad geographic area, with relatively few cases in any one jurisdiction, are much more difficult to detect locally. Rapid analysis of data to detect unusual disease clusters is the first step in recognizing outbreaks. We developed an algorithm for the Public Health Laboratory Information System (PHLIS) (2) that detects unusual clusters by using a statistical quality control method called cumulative sums (CUSUM), a method commonly used in manufacturing. CUSUM has also been applied to medical audits of influenza surveillance in England and Wales (3,4).

### The Algorithm

The statistical problem of detecting unusual disease clusters in public health surveillance is similar to that of detecting clusters of defective items in manufacturing. In both cases, the aim is to detect an unusual number of occurrences. Manufacturing operations use several existing quality control methods, e.g., Shewhart Charts, moving average control, and CUSUM, to indicate abnormalities in data collected (5,6). Of these methods, CUSUM has two unique attributes that make it especially suitable for disease outbreak detection. CUSUM detects smaller shifts from the mean, and it detects similar shifts in the mean more quickly (6-8). The computational simplicity of this method also makes it especially well suited for use on personal computers. Other published methods (9-11) require more personal interactions, e.g., model building, and use more intense computations.

### Applying the Algorithm to Surveillance Data

To evaluate how well the CUSUM algorithm detects unusual clusters of disease, we applied it to the Centers for Disease Control and Prevention (CDC) National *Salmonella* Surveillance System dataset. Since 1962, this surveillance system has collected reports of laboratory-confirmed *Salmonella* isolates from human sources from all U.S. state public health laboratories and the District of Columbia (12). The laboratories serotype clinical isolates of *Salmonella* by the Kauffman-White methods, which subdivide this diverse bacterial genus into more than 2,000 named serotypes (13). Each week, laboratories report to CDC each *Salmonella* strain they have serotyped, along with the age, sex, county of residence of the person from whom it was isolated, and date of specimen collection. The algorithm uses date of specimen collection, which we consider the nearest reliable date to the date the infection began.

A one-sided CUSUM was calculated for every reported *Salmonella* serotype and week by using several values for the expected mean. Different expected means were used in the algorithm to identify which value accurately represented the historical data. First we calculated the mean of 5 weeks and the median of 5 weeks for each *Salmonella* serotype for the same week over the previous 5 years. We then calculated the mean of 15 weeks, which is the mean over a 3-week interval over the past 5 years. For example, for surveillance of the sixth week of 1993, we would use weeks 5 through 7 for each year from 1988 through 1992 to calculate the mean over a 3-week interval. The results of each calculation were compared to identify which value for the expected mean provided the best sensitivity, specificity, and false-positive rate. To minimize the time needed to process the outbreak detection algorithm for each reported serotype for each reported week, the algorithm was processed only for those *Salmonella* serotypes having a potential outbreak, an expected mean greater than zero, and counts greater than the expected mean (Figure 1). Since the entire algorithm is processed when the count for a given serotype exceeds the expected mean, the probability structure of CUSUM is not affected.

### Testing the Algorithm

The outbreak detection algorithm was tested retrospectively to determine how well it discovered known outbreaks. To identify outbreaks, 52 weekly counts were calculated by serotype for each of the reporting sites over 5 years. The algorithm compared $x_t$, the current weekly count of each *Salmonella* serotype reported to the National *Salmonella* Surveillance System, with summary information from the same week over the previous 5 years. The summary information includes $N_t$, the total number of each *Salmonella* serotype reported over the past 5 years for a given week, and the expected mean over the past 5 years for a serotype for a given week. Each week, except week 52, was defined to contain 7 days. The first week of each year included January 1 through January 7; the last week contained 9 days on a leap year and 8 days otherwise.

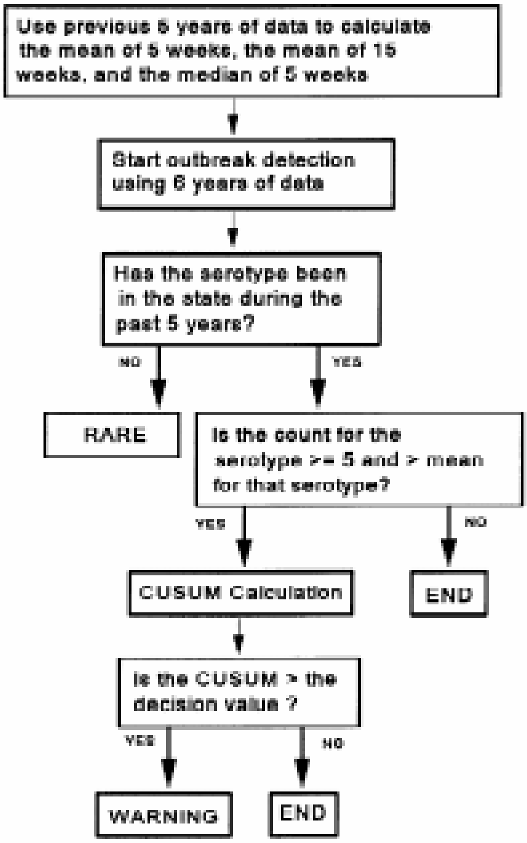

Figure 1. Algorithm for outbreak detection for one serotype for 1 week.[a]

[a]Since we are interested in detecting only increases in the number of isolates of *Salmonella* serotypes, we based our algorithm on a one-sided CUSUM. The numbers vary by serotype, and we assume the numbers of individual serotypes to be normally distributed for any given week in the past 5 years. A one-sided CUSUM determines a positive shift from the expected mean. The

CUSUM ($S_t$) is $S_t = \max(0, S_{t-1} + (z_t - k))$

where $z_t = (\overline{X}_t - \mu_0)/(\sigma/\sqrt{N_t})$, $S_0 = 0$, and $k > 0$.

This simplifies to $S_t = \max(0, S_{t-1} + \frac{\overline{X}_t - (\mu_0 + k\sigma_{\overline{X}_t})}{\sigma_{\overline{X}_t}})(8)$.

The standard deviation was used in our calculations instead of the standard error. $S_t$ cumulates both the positive deviations of counts greater than k standard deviations from the mean and zero for the negative deviation of counts (8,10,14). The central reference, k, determines how many standard deviations are added to the mean. Setting k=1 helped control the variability in counts due to reporting errors, seasonality, and outbreaks.

To detect any count above delta standard deviations from the mean, a CUSUM decision value, h, was set to ensure an appropriate average run length (ARL). The values h=0.5, k=1, and delta=0.5 yielded an ARL=6 years. This ARL allowed consideration of 5 past years of counts and the count for the current year before the CUSUM signals become out of control (15,16).

A rare or uncommon serotype, i.e., a serotype that had not been reported from a state during the past 5 years, was flagged immediately as a serotype of interest. We compared flags generated by the algorithm by state and week with occurrences of reported outbreaks. We considered the sensitivity, specificity, and false-positive rate for three outbreak sizes: 1) any isolates, 2) at least three isolates, and 3) at least five isolates. Data were limited to reports during 1993 and, because we had information about previously reported outbreaks involving this serotype, CDC's *Salmonella* serotype Enteritidis (SE) Outbreak Surveillance System (17). Sensitivity was calculated as the number of outbreaks flagged by the algorithm that matched SE outbreaks reported to CDC by state and by week. Because an outbreak could have received several flags corresponding to different weeks, flags in consecutive weeks were counted as both being correct. Specificity was defined as the number of weeks without flags that corresponded to weeks without reported outbreaks. The false-positive rate was defined as the proportion of flags that did not correspond to outbreaks.

### Results of the Test

The SE Outbreak Surveillance System had 63 outbreaks reported during 1993 from 20 states and one U.S. territory. Of these 63 outbreaks, 38 reports included date of collection. Two of the reported 38 SE outbreaks occurred in the same state in the same week, and multiple outbreaks occurred 1 week apart in the same state. Therefore, it is difficult to distinguish all 38 reported outbreaks as individual outbreaks.

When we used the mean of 5 weeks as the expected mean in the algorithm, 35 states had 230 flags for clusters with ≥3 isolates (Table 1). For clusters of ≥5 isolates, 25 states had 121 flags. Sensitivity calculations on these flags were 0% (0/1) to 100%, specificity was 64% to 100%, and the overall false-positive rate was 77% (Table 2).

When the median of 5 weeks was used for the expected mean in the algorithm, the algorithm flagged SE in 35 states with 380 unusual clusters with ≥3 isolates. Twenty-five states had 210 flags with ≥5 isolates (these states were the same ones that were flagged when the mean of 5 weeks and counts of ≥5 isolates were used). In each instance in which using the median of 5 weeks resulted in an unusual cluster being flagged that had not been flagged using the mean of 5 weeks, the median of 5 weeks was smaller than the mean of 5 weeks. Clusters flagged by using the median of 5 weeks but not flagged by using the mean of 5 weeks were three to 37 isolates, with a mean of seven per cluster. Three of these clusters with five or more isolates were known outbreaks. Thus, using the median of 5 weeks would have detected three more outbreaks than using the mean of 5 weeks, but at the expense of lower specificity.

Evaluating the algorithm by using the mean of 15 weeks for the expected mean, we found 125 SE flags in 25 states on clusters with ≥5 isolates. These were the same states flagged when the mean of 5 weeks was used for the expected mean. Each time a flag occurred using the mean of 15 weeks, while no corresponding flag occurred using the mean of 5 weeks, the mean of 15 weeks was smaller than the mean of 5 weeks. In this scenario, the sizes of the clusters were 3 to 8 isolates, with an average of 5 isolates per cluster. In comparison, the mean of 5 weeks was associated with a higher specificity than the mean of 15 weeks.

Without a way to calculate an overall specificity for all serotypes, the decision about which value to use as the expected mean in the algorithm was based on the data gathered about SE. Using the median of 5 weeks produced the largest number of flags and the lowest specificity; a mean of 15 weeks generated the second highest number of flags and the second lowest specificity; and using the mean of 5 weeks produced the fewest flags and the highest specificity. Even though using both the median of 5 weeks or the mean of 15 weeks produced additional early flags, this negligible increase in sensitivity was associated with a decrease in specificity. Therefore, we elected to use the mean of 5 weeks for the expected mean in the algorithm, to obtain the highest specificity.

### An Assessment of the Algorithm

The CUSUM algorithm provides a simple method to evaluate surveillance data as they are being gathered and provides sensitive and rapid identification of unusual clusters of disease. In this algorithm, a mean of 5 weeks was a better value for the expected mean than a median of 5 weeks or a mean of 15 weeks. Using a mean of 5 weeks, the algorithm failed to flag reported outbreaks only three times. In addition, a median of 5 weeks and a mean of 15 weeks were associated

Table 1. Number of flags produced for *Salmonella* serotype Enteritidis, 1993

| State | All Isolates | | | Three or more isolates | | | Five or more isolates | | |
|---|---|---|---|---|---|---|---|---|---|
| | Mean 5 wks | Mean 15 wks | Median 5 wks | Mean 5 wks | Mean 15 wks | Median 5 wks | Mean 5 wks | Mean 15 wks | Median 5 wks |
| Alaska | 3 | 3 | 3 | 0 | 0 | 0 | 0 | 0 | 0 |
| Arizona | 12 | 13 | 18 | 4 | 4 | 4 | 1 | 1 | 1 |
| Arkansas | 3 | 3 | 3 | 0 | 0 | 0 | 0 | 0 | 0 |
| Colorado | 21 | 23 | 35 | 13 | 13 | 15 | 1 | 1 | 1 |
| Connecticut | 12 | 13 | 31 | 11 | 12 | 26 | 9 | 10 | 21 |
| Delaware | 7 | 8 | 12 | 1 | 1 | 2 | 0 | 0 | 0 |
| District of Columbia | 4 | 4 | 8 | 1 | 1 | 1 | 0 | 0 | 0 |
| Florida | 15 | 15 | 15 | 1 | 1 | 1 | 1 | 1 | 1 |
| Georgia | 2 | 2 | 2 | 2 | 2 | 2 | 0 | 0 | 0 |
| Hawaii | 7 | 7 | 7 | 0 | 0 | 0 | 0 | 0 | 0 |
| Idaho | 12 | 12 | 14 | 0 | 0 | 0 | 0 | 0 | 0 |
| Illinois | 12 | 12 | 23 | 12 | 12 | 23 | 12 | 12 | 23 |
| Indiana | 22 | 22 | 33 | 14 | 14 | 17 | 7 | 7 | 8 |
| Iowa | 10 | 11 | 16 | 5 | 5 | 5 | 1 | 1 | 1 |
| Kansas | 12 | 12 | 16 | 1 | 1 | 1 | 1 | 1 | 1 |
| Kentucky | 10 | 10 | 13 | 0 | 0 | 0 | 0 | 0 | 0 |
| Louisiana | 16 | 17 | 18 | 7 | 7 | 7 | 0 | 0 | 0 |
| Maryland | 8 | 9 | 25 | 8 | 9 | 25 | 4 | 5 | 20 |
| Massachusetts | 5 | 5 | 9 | 5 | 5 | 9 | 5 | 5 | 9 |
| Michigan | 11 | 11 | 34 | 9 | 9 | 22 | 7 | 7 | 9 |
| Minnesota | 20 | 24 | 31 | 17 | 19 | 20 | 6 | 6 | 6 |
| Missouri | 17 | 19 | 30 | 5 | 5 | 6 | 1 | 1 | 1 |
| Nevada | 6 | 6 | 6 | 2 | 2 | 2 | 1 | 1 | 1 |
| New Hampshire | 19 | 22 | 23 | 3 | 3 | 3 | 0 | 0 | 0 |
| New Jersey | 7 | 7 | 25 | 7 | 7 | 25 | 7 | 7 | 24 |
| New Mexico | 14 | 14 | 17 | 7 | 7 | 7 | 4 | 4 | 4 |
| New York | 11 | 12 | 28 | 10 | 11 | 26 | 7 | 7 | 18 |
| North Dakota | 7 | 7 | 8 | 0 | 0 | 0 | 0 | 0 | 0 |
| Ohio | 11 | 13 | 25 | 10 | 12 | 19 | 7 | 8 | 11 |
| Oklahoma | 3 | 3 | 3 | 0 | 0 | 0 | 0 | 0 | 0 |
| Oregon | 16 | 17 | 21 | 4 | 4 | 4 | 0 | 0 | 0 |
| Pennsylvania | 1 | 1 | 1 | 1 | 1 | 1 | 1 | 1 | 1 |
| Rhode Island | 18 | 18 | 22 | 7 | 7 | 8 | 2 | 2 | 2 |
| South Carolina | 10 | 10 | 13 | 6 | 6 | 6 | 1 | 1 | 1 |
| South Dakota | 15 | 15 | 18 | 1 | 1 | 1 | 0 | 0 | 0 |
| Tennessee | 4 | 4 | 14 | 3 | 3 | 6 | 0 | 0 | 0 |
| Texas | 15 | 16 | 24 | 9 | 9 | 10 | 5 | 5 | 5 |
| Utah | 13 | 14 | 16 | 2 | 2 | 2 | 0 | 0 | 0 |
| Vermont | 28 | 29 | 30 | 15 | 15 | 16 | 9 | 9 | 10 |
| Virginia | 12 | 13 | 37 | 12 | 13 | 32 | 8 | 9 | 16 |
| West Virginia | 3 | 3 | 5 | 1 | 1 | 1 | 0 | 0 | 0 |
| Wisconsin | 14 | 15 | 31 | 14 | 14 | 25 | 13 | 13 | 15 |
| Total | 468 | 494 | 763 | 230 | 238 | 380 | 121 | 125 | 210 |

with lower specificity than the mean of 5 weeks. Therefore, to achieve the best specificity we used a mean of 5 weeks.

The sensitivity, specificity, and false-positive rate results indicate that the algorithm works well. However, there are several potential limitations to calculating sensitivity, specificity, and the false-positive rate as we did. Some of these include outbreak size, lack of reporting of isolates, duplicate isolate reports, and under-reporting of outbreaks. Constraints on public health resources may limit investigation of small outbreaks of SE. Therefore, we did not include these in the calculation of sensitivity. Under-reporting of isolates could cause the algorithm to miss an outbreak, regardless of its size. Under-reporting of known SE outbreaks could also inflate our estimates of specificity.

Table 2. Sensitivity and specificity for 1993 of the *Salmonella* outbreak detection algorithm and of known outbreaks of *Salmonella* serotype Enteritidis

| ST | Sensitivity no. flagged wks/ no. outbreaks (%) | | | Specificity no. nonflagged wks/ no. possible nonflagged wks (%) | | | False-positive ($P_{f+}$) nonoutbreak flags/ no. flags (%) | | |
|---|---|---|---|---|---|---|---|---|---|
| | Mean 5 wks | Mean 15 wks | Median 5 wks | Mean 5 wks | Mean 15 wks | Median 5 wks | Mean 5 wks | Mean 15 wks | Median 5 wks |
| AK | 0/0 | 0/0 | 0/0 | 52/52 (100) | 52/52 (100) | 52/52 (100) | | | |
| AZ | 0/0 | 0/0 | 0/0 | 51/52 (98) | 51/52 (98) | 51/52 (98) | 1/1 (1) | 1/1 (1) | 1/1 (1) |
| AR | 0/0 | 0/0 | 0/0 | 52/52 (100) | 52/52 (100) | 52/52 (100) | | | |
| CO | 0/0 | 0/0 | 0/0 | 51/52 (98) | 51/52 (98) | 51/52 (98) | 1/1 (1) | 1/1 (1) | 1/1 (1) |
| CT | 5/6 (83) | 5/6 (83) | 5/6 (83) | 39/46 (85) | 39/46 (85) | 36/46 (78) | 4/9 (44) | 5/10 (50) | 16/21 (76) |
| DE | 0/0 | 0/0 | 0/0 | 52/52 (100) | 52/52 (100) | 52/52 (100) | | | |
| DC | 0/0 | 0/0 | 0/0 | 52/52 (100) | 52/52 (100) | 52/52 (100) | | | |
| FL | 0/1 | 0/1 | 0/1 | 51/52 (98) | 51/52 (98) | 51/52 (98) | 1/1 (1) | 1/1 (1) | 1/1 (1) |
| GA | 0/0 | 0/0 | 0/0 | 52/52 (100) | 52/52 (100) | 52/52 (100) | | | |
| HI | 0/0 | 0/0 | 0/0 | 52/52 (100) | 52/52 (100) | 52/52 (100) | | | |
| ID | 0/0 | 0/0 | 0/0 | 52/52 (100) | 52/52 (100) | 52/52 (100) | | | |
| IL | 1/1 (100) | 1/1 (100) | 1/1 (100) | 16/25 (64) | 16/25 (64) | 6/25 (24) | 11/12 (92) | 11/12 (92) | 22/23 (96) |
| IN | 0/0 | 0/0 | 0/0 | 45/52 (87) | 45/52 (87) | 44/52 (85) | 7/7 (1) | 7/7 (1) | 8/8 (1) |
| IA | 1/1 (100) | 1/1 (100) | 1/1 (100) | 51/51 (100) | 51/51 (100) | 51/51 (100) | 0/1 (0) | 0/1 (0) | 0/1 (0) |
| KS | 1/1 (100) | 1/1 (100 ) | 1/1 (100) | 51/51 (100) | 51/51 (100) | 51/51 (100) | 0/1 (0) | 0/1 (0) | 0/1 (0) |
| KY | 0/0 | 0/0 | 0/0 | 52/52 (100) | 52/52 (100) | 52/52 (100) | | | |
| LA | 0/0 | 0/0 | 0/0 | 52/52 (100) | 52/52 (100) | 52/52 (100) | | | |
| MD | 3/4 (75) | 3/4 (75) | 4/4 (100) | 44/48 (92) | 43/48 (90) | 31/48 (65) | 1/4 (25) | 2/5 (40) | 16/20 (80) |
| MA | 0/0 | 0/0 | 0/0 | 47/52 (90) | 47/52 (90) | 43/52 (83) | 5/5 (1) | 5/5 (1) | 9/9 (1) |
| MI | 0/0 | 0/0 | 0/0 | 45/52 (87) | 45/52 (87) | 43/52 (83) | 7/7 (1) | 7/7 (1) | 9/9 (1) |
| MN | 0/0 | 0/0 | 0/0 | 46/52 (88) | 46/52 (88) | 46/52 (88) | 6/6 (1) | 6/6 (1) | 6/6 (1) |
| MO | 0/0 | 0/0 | 0/0 | 51/52 (98) | 51/52 (98) | 51/52 (98) | 1/1 (1) | 1/1 (1) | 1/1 (1) |
| NV | 0/0 | 0/0 | 0/0 | 51/52 (98) | 51/52 (98) | 51/52 (98) | 1/1 (1) | 1/1 (1) | 1/1 (1) |
| NH | 0/0 | 0/0 | 0/0 | 52/52 (100) | 52/52 (100) | 52/52 (100) | | | |
| NJ | 1/2(50) | 1/2(50) | 2/2 (100) | 44/50 (88) | 44/50 (88) | 28/50 (56) | 6/7 (86) | 6/7 (86) | 22/24 (92) |
| NM | 0/0 | 0/0 | 0/0 | 48/52 (92) | 48/52 (92) | 48/52 (92) | 4/4 (1) | 4/4 (1) | 4/4 (1) |
| NY | 7/10 (70) | 7/10 (70) | 8/10 (80) | 41/42 (98) | 40/42 (95) | 35/42 (83) | 7/7 (0) | 7/7 (0) | 10/18 (56) |
| ND | 0/0 | 0/0 | 0/0 | 52/52 (100) | 52/52 (100) | 52/52 (100) | | | |
| OH | 0/0 | 0/0 | 0/0 | 45/52 (87) | 44/52 (85) | 41/52 (79) | 7/7 (1) | 8/8 (1) | 11/11 (1) |
| OK | 0/0 | 0/0 | 0/0 | 52/52 (100) | 52/52 (100) | 52/52 (100) | | | |
| OR | 0/0 | 0/0 | 0/0 | 52/52 (100) | 52/52 (100) | 52/52 (100) | | | |
| PA | 1/1 (100) | 1/1 (100) | 1/1 (100) | 3/3 (100) | 3/3 (100) | 3/3 (100) | 0/1 (0) | 0/1 (0) | 0/1 (0) |
| RI | 0/0 | 0/0 | 0/0 | 50/52 (96) | 50/52 (96) | 50/52 (96) | 2/2 (1) | 2/2 (1) | 2/2 (1) |
| SC | 1/1 (100) | 1/1 (100) | 1/1 (100) | 51/51 (100) | 51/51 (100) | 51/51 (100) | 1/1 (0) | 1/1 (0) | 1/1 (0) |
| SD | 0/0 | 0/0 | 0/0 | 52/52 (100) | 52/52 (100) | 52/52 (100) | | | |
| TN | 0/0 | 0/0 | 0/0 | 52/52 (100) | 52/52 (100) | 52/52 (100) | | | |
| TX | 2/2 (100) | 2/2 (100) | 2/2 (100) | 48/50 (96) | 48/50 (96) | 48/50 (96) | 3/5 (60) | 3/5 (60) | 3/5 (60) |
| UT | 0/0 | 0/0 | 0/0 | 52/52 (100) | 52/52 (100) | 52/52 (100) | | | |
| VT | 5/6 (83) | 5/6 (83) | 5/6 (83) | 43/46 (93) | 43/46 (93) | 43/46 (93) | 4/9 (44) | 4/9 (44) | 5/10 (50) |
| VA | 0/1 (0) | 0/1 (0) | 0/1 (0) | 43/51 (84) | 43/51 (84) | 35/51 (69) | 8/8 (1) | 9/9 (1) | 16/16 (1) |
| WV | 0/0 | 0/0 | 0/0 | 52/52 (100) | 52/52 (100) | 52/52 (100) | | | |
| WI | 1/1 (100) | 1/1 (100) | 1/1 (100) | 40/51 (78) | 40/51 (78) | 38/51 (75) | 12/13 (92) | 12/13 (92) | 14/15 (93) |
| Total | 29/38 (76) | 29/38 (76) | 32/38 (82) | 1978/2072 (95) | 1975/2072 (95) | 1909/2072 (92) | 92/121 (76) | 96/125 (77) | 178/210 (85) |

An outbreak detection algorithm must have high specificity (i.e., few false flags). The algorithm can be adjusted to achieve better specificity, which would benefit state health departments that may choose to investigate small clusters.

Seasonal shifts in the incidence of *Salmonella* can interfere with the sensitivity of the outbreak detection algorithm. In our study, we examined only unusual clusters of *Salmonella* that were above the normal seasonal patterns. Thus, we may have missed smaller outbreaks that were obscured by seasonality. For example, we could have overlooked an outbreak of three cases if it occurred in a season with a high background number of reported cases.

The ability of the algorithm to detect outbreaks rapidly is also affected by the speed with which serotyping is done and the results reported by state public health laboratories.

In early spring 1995, we implemented the algorithm on a weekly basis, looking for unusual clusters at the state, regional, and national levels among *Salmonella* isolate data reported each week from state public health laboratories to CDC. An international outbreak of *Salmonella* serotype Stanley was flagged in May 1995 (Figure 2). *S*. Stanley is an unusual serotype in the United States, with only 219 cases reported in 1994. The ensuing epidemiologic investigation

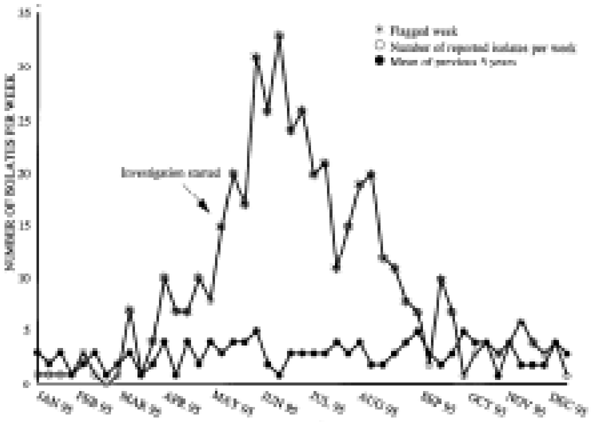

Figure 2. *Salmonella* outbreak detection algorithm *Salmonella* serotype Stanley isolates, United States, 1995.

implicated alfalfa sprouts as the vehicle of infection (18). Rapid detection of this outbreak concluded in identification of a new vehicle of salmonellosis and prompted development of prevention measures.

**L. C. Hutwagner, E. K. Maloney, N. H. Bean, L. Slutsker, and S. M. Martin**
Centers for Disease Control and Prevention,
Atlanta, Georgia, USA

### References

1. Centers for Disease Control and Prevention. Addressing emerging infectious disease threats, a prevention strategy for the United States. Atlanta: CDC, 1994.
2. Martin SM, Bean NH. Data management issues for emerging diseases and new tools for managing surveillance and laboratory data. Emerging Infect Dis 1995;1:124-8.
3. Williams SM, Parry BR, Schlup MMT. Quality control: an application of the CUSUM. BMJ 1992;304:1359-61.
4. Tillett HE, Spencer IL. Influenza surveillance in England and Wales using routine statistics. Journal of Hygiene 1982;88:83-94.
5. Montgomery DC. Introduction to statistical quality control. New York: John Wiley and Sons; 1985.
6. Banks J. Principles of quality control. New York: John Wiley and Sons; 1989.
7. Lucas JM. The design and use of V-Mask control schemes. Journal of Quality Technology 1976;8:1-12.
8. Lucas JM. Counted data CUSUM's. Technometrics 1985;27:129-44.
9. Stroup DF, Williamson GD, Herndon JL. Detection of aberrations in the occurrence of notifiable diseases surveillance data. Stat Med 1989;8:323-9.
10. Watier L, Richardson S. A time series construction of an alert threshold with application to *S. bovismorbificans* in France. Stat Med 1991;10:1493-509.
11. Farringtion CP, Andrews NJ, Beale AD, Catchpole MA. A statistical algorithm for the early detection of outbreaks of infectious disease. Journal of the Royal Statistical Society Series A 1996;159:547-63.
12. Centers for Disease Control and Prevention. Salmonella surveillance. 1993-1995 Annual Tabulation Summary. Atlanta: CDC; 1996.
13. McWhorter-Murlin AC, Hickman-Brenner FW. Identification and serotyping of *Salmonella* and an update of the Kauffmann-White Scheme. Atlanta: CDC; 1994.
14. SAS Institute Inc. SAS/QC software: reference, version 6. 1st ed. Cary (NC): SAS Institute Inc.; 1989.
15. Goel AL, Wu SM. Determination of A.R.L. and a contour nomogram for CUSUM charts to control normal mean. Technometrics 1971;13:221-30.
16. Lucas JM, Crosier RB. Fast initial response for CUSUM quality-control schemes: give your CUSUM a head start. Technometrics 1982;24:199-205.
17. Mishu B, Koeher J, Lee AL, Rodrigue D, Brenner FH, Blake P, Tauxe R. Outbreaks of *Salmonella enteritidis* infections in the United States, 1985-1991. J Infect Dis 1994;169:547-52.
18. Mahon B, Ponka A, Hall W, Komatsu K, Dietrich S, Siitonen A, et al. An international outbreak of *Salmonella* infections caused by alfalfa sprouts grown from contaminated seed. J Infect Dis 1997;175:876-82.
